# Variables Associated with Performance of an Active Limb Movement following Within-Session Instruction in People with and People without Low Back Pain

**DOI:** 10.1155/2013/867983

**Published:** 2013-08-01

**Authors:** Sara A. Scholtes, Barbara J. Norton, Sara P. Gombatto, Linda R. Van Dillen

**Affiliations:** ^1^Program in Physical Therapy, Saint Louis University, 3437 Caroline St., St. Louis, MO 63104, USA; ^2^Program in Physical Therapy, Washington University School of Medicine, St. Louis, MO 63108, USA; ^3^Department of Neurology, Washington University School of Medicine, St. Louis, MO 63110, USA; ^4^Program in Physical Therapy, San Diego State University, San Diego, CA 92182, USA; ^5^Department of Orthopaedic Surgery, Washington University School of Medicine, St. Louis, MO 63110, USA

## Abstract

Modification of a movement pattern can be beneficial in decreasing low back pain (LBP) symptoms. There is variability, however, in how well people are able to modify performance of a movement. What has not been identified is the factors that may affect a person's ability to modify performance of a movement. We examined factors related to performance of active hip lateral rotation (HLR) following standardized instructions in people with and people without LBP. Data were collected during performance of HLR under 3 conditions: passive, active, and active instructed. In people with LBP, motion demonstrated during the passive condition (*r* = 0.873, *P* < 0.001), motion demonstrated during the active condition (*r* = 0.654, *P* = 0.008), and gender (*r* = 0.570, *P* = 0.027) were related to motion demonstrated during the active-instructed condition. Motion demonstrated during the passive condition explained 76% (*P* < 0.001) of the variance in motion demonstrated during the active-instructed condition. A similar relationship did not exist in people without LBP. The findings of the study suggest that it may be important to assess motion demonstrated during passive HLR to determine how difficult it will be for someone with LBP to modify the performance of HLR. Prognosis should be worst for those who display similar movement patterns during passive HLR and active-instructed HLR.

## 1. Introduction

Low back pain (LBP) is a highly prevalent, very costly, and extremely complex condition [1, 2]. Consistent with the view that LBP is multifactorial in nature, researchers have studied relationships between LBP and many potentially relevant factors in many domains [1, 2]. Yet, LBP continues to be a leading cause of activity limitation [1, 2]. Clearly, key factors contributing to LBP remain to be identified. 

One factor that may contribute to the development and persistence of LBP is the specific way in which individuals with LBP move when they perform tasks throughout the day. Because performance of everyday tasks seldom requires people to use the full available range of motion at specific joints [3, 4], it is likely that the way movements are performed within small ranges of movement can create problems. For example, if elements of the lumbopelvic region (e.g., pelvis and lumbar vertebrae) begin to move soon after a person begins a simple task like hip rotation, then rotating the hip even a small amount during daily activities may increase the frequency of movement in the lumbopelvic region. The increased frequency of movement could increase stress on tissues in the lumbopelvic region, particularly if the movements are repeated frequently throughout the day [5]. Over time and with more repetitions of the motion, the accumulation of tissue stress in the lumbopelvic region may be greater than the adaptive tissue remodeling necessary to prevent tissue failure. If so, tissue failure may occur and LBP symptoms may result [5–8]. 

Some details about how people move during different tasks have been investigated. Several investigators have reported that there are differences between people with and people without LBP in the timing of motion between (1) different elements within the lumbopelvic region and (2) an adjacent limb region (e.g., hip) during a number of different tasks [9–13]. In addition, we have reported that (1) modifying the timing of motion between adjacent regions during tasks which involve limb movement decreases LBP symptoms [14, 15], (2) people with and people without LBP are able to modify how they perform a limb movement task following within-session instruction [16], and (3) compared to a nonspecific intervention protocol, a 6-week movement-specific intervention protocol results in delayed and decreased movement of elements within the lumbopelvic region during limb movement tasks [17]. Although recent evidence suggests that movement of elements within the lumbopelvic region during limb movement tasks may be related to LBP and is modifiable, what remains to be explored is which factors are related to how an individual performs a motion following instruction. 

Recently we reported that people with and people without LBP were able to modify performance of hip lateral rotation (HLR) following standardized instructions. People with and people without LBP were able to complete a greater amount of HLR prior to the onset of motion in the lumbopelvic region after receiving instructions to keep the pelvis stable during HLR [16]. However, despite providing standardized instructions to all participants, corrections following instruction ranged from complete elimination of rotation by all elements of the lumbopelvic region during HLR to little, if any, correction. Thus, we seek to identify factors that might explain the differences in performance between individuals. 

Many factors may be related to how an individual performs a task. Gender, joint hypermobility, and anthropometrics previously have been identified as factors that may affect performance of different tasks [18–26]. Because factors such as gender, joint hypermobility, and anthropometrics may affect how an individual performs a task, they also may affect performance of a task following instruction. Additionally, because gender, joint hypermobility, and anthropometrics are inherent to the person and do not change between testing conditions, we assume that, if these factors affect movement, performance of a task prior to instruction will be related to performance of a task following instruction. 

Passive tissue characteristics also may be related to performance of a task before and after instruction. Relative stiffness, defined as a difference in stiffness between adjacent body regions, has been proposed to explain why motion occurs in one region when an adjacent region moves during an active or a passive task [27, 28]. The region that is thought to be less stiff is more likely to move during a task. For example, if elements within the lumbopelvic region are less stiff than the hip region, rotation is likely to occur in the lumbopelvic region almost as soon as the hip moves during active or passive HLR. Although the factors noted are presumed to be related to how a person performs or modifies a task following instruction, direct evidence is not available. 

The purpose of the current study was to examine factors presumed to be associated with performance of active HLR following standardized, within-session instruction. Performance of hip lateral rotation while prone was examined in the current study because (1) active HLR can provoke symptoms in people with LBP [24, 29] and (2) people are able to modify HLR following within-session instruction [16]. Hip lateral rotation was examined under three conditions, *active, active instructed, *and *passive*. During the *active *condition, subjects performed HLR without any specific instructions about the lumbopelvic region in order to capture the natural movement pattern of the hip and elements of the lumbopelvic region during active HLR. During the *active-instructed *condition, subjects performed HLR after receiving instructions intended to eliminate rotation of elements in the lumbopelvic region during active HLR. During the *passive* condition, subjects were moved passively through the range of HLR to assess the movement pattern of the hip and elements of the lumbopelvic region during passive HLR. For people with and people without LBP, we hypothesized that the motion displayed during (1) active HLR performed prior to instruction and (2) passive HLR would be related to performance of active HLR following instruction. 

A better understanding of factors related to the performance of a particular task following instruction could be important for determining a patient's plan of care and prognosis. Some factors, such as flexibility, are modifiable and may be addressable with intervention. Other factors, such as height, are not modifiable but may still be addressable through compensatory intervention strategies. Still other factors may not be modifiable or addressable. A patient with less addressable factors may have a poorer prognosis. Thus, identifying factors that are related to performance of a task following instruction may help a clinician tailor a plan of care and determine the most appropriate prognosis for a patient.

## 2. Materials and Methods

### 2.1. Subjects

Nineteen people with LBP (LBP group) and 20 people without LBP (NoLBP group) were included in the current study. People were included in the LBP group if they reported having chronic or recurrent [30] LBP for at least 6 months. People were excluded from the NoLBP group if they reported having experienced LBP that limited daily activities for more than 3 days or for which they sought medical attention. Potential subjects were excluded from either group if they reported having (1) a body mass index greater than 30, (2) a hip or knee injury that limited daily activities, (3) a history of a spinal fracture or surgery, or (4) a diagnosis of a spinal structural deformity, systemic inflammatory condition, or other serious medical condition that could affect movement (e.g., Parkinson's disease). Prior to participation in the study, all subjects read and signed an informed consent approved by the university's Human Research Protection Office. 

Following completion of informed consent, all subjects completed self-report questionnaires before donning fitted shorts (all subjects) and a sports bra (female subjects) for the remainder of testing. Subjects then completed the laboratory measures followed by the clinical tests. Specific methods for each of these items are described in more detail below. 

### 2.2. Self-Report Questionnaires

All subjects completed a demographic and LBP history questionnaire. Subjects with LBP also completed a modified Oswestry Disability Index [31] and provided a current verbal numeric pain rating score [32].

### 2.3. Laboratory Measures

#### 2.3.1. Kinematic Data

Kinematic data were collected using a 6-camera motion capture system (EVaRT, Motion Analysis Corporation, Santa Rosa, CA, USA). Prior to data collection, reflective markers were placed over landmarks of the trunk, pelvis, and lower limbs to capture motion during testing. Markers were adhered to the skin superficial to C7 and bilateral lateral malleoli, lateral knee joint lines, greater trochanters, and posterior superior iliac spines (PSISs). 

Kinematic data were collected while subjects performed HLR under 3 conditions: *active*, *active instructed, *and *passive*. For all trials, subjects were positioned in prone on a portable massage table with their head in midline and their arms at their side. The tested lower limb was positioned with the knee flexed to 90° and with the hip in neutral abduction/adduction. Prior to the start of each trial, the examiner held the hip of the tested limb in 5° of medial rotation to allow the subject to relax. During all *active *and *active-instructed* trials, subjects moved at a self-selected speed. The subjects were instructed to “bring the foot in as far as possible,” that is, lateral rotation, and then “return the foot to the start position,” that is, medial rotation. For *active*-*instructed* HLR trials, subjects were provided additional instruction intended to eliminate motion of all elements within the lumbopelvic region during HLR. Prior to each *active-instructed* trial, the subjects were told to contract the abdominal muscles and to not allow the pelvis to rotate during HLR; the tester simultaneously provided tactile cues on the abdominal muscles and posterior pelvis. No verbal or tactile cueing was provided during the *active-instructed* trials. Prior to each *passive* trial, the subject was instructed to relax while the tester performed maximal HLR and then returned to the start position. End of motion during the *passive* trials was defined as the point in the range at which either (1) the test was unable to rotate the hip any further or (2) the shank of the tested limb contacted the nontested limb. No external stabilization (e.g., belt) of the pelvis was provided during the *active, active-instructed, *or *passive* HLR trials. 

All subjects completed the HLR trials in the following order: *active, active instructed*, and *passive. *The *active* and *active-instructed* trials were completed prior to *passive* trials so that the tester's movement of the hip during the *passive* trials would not affect the subject's performance during either the *active *or the* active-instructed* condition. The side tested first (right and left) was randomized within each condition. Five trials of HLR were performed with the right and left hips separately for both the *active* and *passive* conditions; ten trials were performed with the right and left hips separately for the *active-instructed* condition. 

#### 2.3.2. Electromyographic Data

Electromyographic (EMG) data were collected to assess muscle activity during the *passive* trials using a Myosystem 1400 A (Noraxon, Inc., Scottsdale, AZ, USA). Muscle activity was recorded bilaterally from the following muscles: latissimus dorsi [33], lumbar erector spinae [34], multifidus [35], rectus abdominus [34], internal oblique [34], external oblique, and lateral hamstrings [36] during the *passive* trials. Subjects were instructed to remain relaxed throughout the *passive *trials. 

### 2.4. Clinical Tests

Femoral anteversion, measured with methods described by Ruwe et al. [37], and passive HLR range of motion, measured with an inclinometer, were assessed bilaterally with the subject in prone and the knee flexed to 90°. To minimize pelvic motion during measures of passive HLR, the subject's pelvis was stabilized with a belt and the assistance of a second tester. Generalized joint hypermobility was tested using the Beighton Hypermobility Scale [38].

### 2.5. Data Processing

#### 2.5.1. Kinematic Data

Kinematic data were collected at a sampling rate of 60 Hz and were initially filtered using a fourth order dual-pass, butterworth filter with a cut-off frequency of 2.5 Hz. After initial filtering, the start and end points of HLR and selected elements of lumbopelvic motion were determined, and movement time was calculated. Because subjects were allowed to move at self-selected speeds, the raw data was refiltered using a subject-specific cut-off frequency (fc_*ss*_) [39] that was calculated by taking the reciprocal of 15% of the period, fc_*ss*_ = 1/0.15 ∗ (2 ∗ movement time) [24]. 

Spine length was defined as the distance between C7 and the midpoint between the PSIS markers. Shank length was defined as the distance between knee joint line and the lateral malleolus.

**Figure 1:**
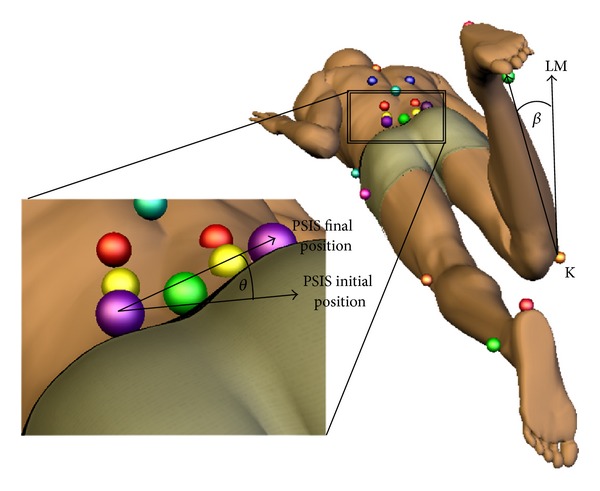
Kinematic model with hip lateral rotation (*β*) and lumbopelvic rotation (*θ*) calculations. LM: lateral malleolus, K: knee, PSIS: posterior superior iliac spine. Reprinted from *Manual Therapy *[16].

Hip lateral rotation was calculated using a lower limb vector defined by the lateral malleolus and the lateral knee joint line markers. Hip lateral rotation was calculated as a change in the angle of the lower limb vector relative to the initial position [24] (Figure 1). The start and end of HLR during each trial were defined as the point at which angular velocity of the lower leg vector exceeded 5% and 99.5% of the maximal angular velocity, respectively.

Rotation of elements within the lumbopelvic region (lumbopelvic rotation) was captured using a pelvic vector defined by the PSIS markers. Lumbopelvic rotation was calculated as a change in angle of the pelvic vector relative to the initial position (Figure 1). The start and end of lumbopelvic rotation were defined as the point at which angular velocity of the pelvic vector exceeded 10% and 99.5% of the maximal angular velocity, respectively. 

**Figure 2:**
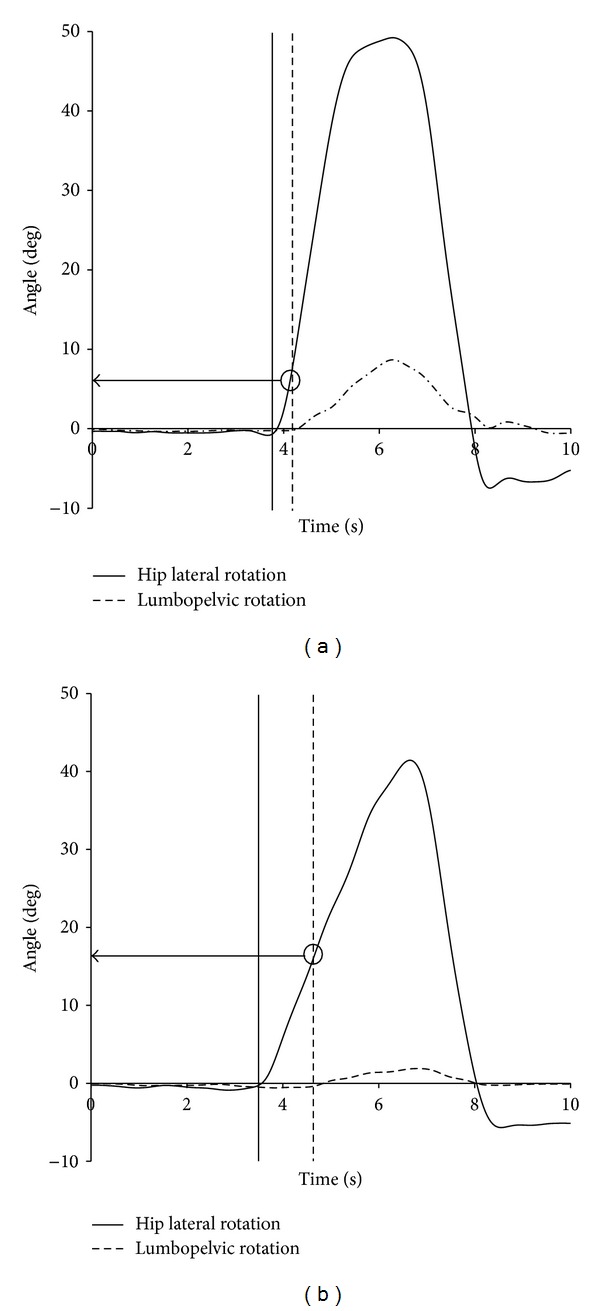
Relative motion index (RMI) was defined as the amount of hip lateral rotation angular motion completed prior to the start of lumbopelvic rotation. The vertical lines indicate the start of hip lateral rotation and lumbopelvic rotation. (a) An example of a smaller RMI. (b) An example of a larger RMI.

A relative motion index (RMI) was calculated to assess indirectly the relative stiffness between the elements of the lumbopelvic region and the hip region. The RMI was defined as the amount of HLR angular motion completed prior to the start of lumbopelvic rotation (Figure 2). A small RMI would indicate greater stiffness of the hip region relative to the elements within the lumbopelvic region. A large RMI would indicate less stiffness of the hip region relative to the elements within the lumbopelvic region.

#### 2.5.2. Electromyographic Data

Electromyographic data were sampled at a rate of 1200 Hz and were bandpass filtered at 10–500 Hz. Analog data were digitized and synchronized with the kinematic data. Electromyographic data were full-wave rectified and filtered using a fourth order dual-pass, butterworth filter with a filtering frequency of 50 Hz. Baseline EMG activity was obtained for 50 ms prior to the initiation of HLR movement. A muscle was considered active during the *passive *HLR motion if the magnitude of EMG activity exceeded 3 standard deviations above the baseline level [40, 41] and, if so, the trial was eliminated from the data set. Four subjects with LBP were eliminated from the data because muscle activity was detected during all *passive *trials. The final data set included 15 subjects with LBP and 20 subjects without LBP.

### 2.6. Data Analyses

All statistical analyses were performed using IBM SPSS Statistics 19 (SPSS Inc., Chicago, IL, USA). Differences between groups (LBP, NoLBP) for all variables were tested using either a Chi-square or independent samples *t*-test. Values for laboratory measures were based on the average of both limbs for all trials of each condition. Correlational and hierarchical linear regression analyses were performed separately for each group (LBP, NoLBP). Correlations among variables were tested using the Pearson product-moment correlation coefficients. Variables that were correlated significantly (*P* < 0.05) with the criterion variable, RMI during the *active-instructed *condition, were included in a hierarchical linear regression analysis. The order of variable entry was based on theoretical importance. 

## 3. Results

There were no group differences in any subject characteristics (Table 1). 

### 3.1. Low Back Pain Group

The only variables that were correlated significantly with the criterion variable, RMI during the *active-instructed *condition, were (1) RMI during the *passive* condition, (2) RMI during the *active* condition, and (3) gender (Table 2). As the RMI during the *passive *condition and the *active* condition increased, the RMI during the *active-instructed *condition increased. Compared to men, women demonstrated a larger RMI during the *active-instructed* condition. 

The RMI during the *passive *condition, the RMI during the *active *condition, and gender also were correlated positively with each other (Table 3). As RMI during the *passive* condition increased, the RMI during the *active* condition also increased. Compared to men, women demonstrated a larger RMI during both the *passive* and the *active* conditions. None of the correlation coefficients between the criterion variable and any other variables were significant (Table 2).

Variables statistically correlated with the criterion variable, RMI during the *active-instructed* condition, were entered into the regression analysis in the following order: (1) RMI during the *passive *condition, (2) RMI during the *active* condition, and (3) gender. The RMI during the *passive* condition was entered into the regression analysis first because we were most interested in how much variance in the RMI during the *active-instructed* condition would be explained solely by what happens during a passive movement where, presumably, no active factors are involved. The RMI during the *active *condition was entered into the regression analysis second in order to determine if any additional variance was explained by the subjects' natural movement patterns during HLR.

The RMI during the *passive* condition explained 76.2% of the variance in the criterion variable; adding the RMI during the *active* condition and gender did not explain any additional variance (Table 4). 

### 3.2. No LBP Group

The only variable that was correlated significantly with the criterion variable was shank length (Table 2); as shank length decreased, the RMI during the *active-instructed *condition increased. Because only 1 variable was correlated significantly with the criterion variable, a regression analysis was not performed. 

## 4. Discussion

The purpose of the current study was to examine factors associated with performance of HLR following standardized, within-session instruction. In people with LBP, the RMI during the *passive *condition was most strongly related to the RMI during the *active-instructed* condition. People who demonstrate rotation of elements within the lumbopelvic region shortly after the start of HLR during the *passive* conditionalso did so during the *active-instructed *condition. Although gender and the RMI during the *active* condition were correlated with the criterion variable, these two variables did not explain any additional statistically significant variance in the criterion variable. These findings were unique to people with LBP. The only variable correlated with the criterion variable in people without LBP was shank length.

Clinically, therapists assess passive movements as part of a typical musculoskeletal examination. The passive movements often are included to examine characteristics such as structural abnormalities, tissue stiffness, end-range extensibility of a joint, pain behavior, and radicular symptoms [42–45]. In patients with LBP, a clinician may perform a passive straight leg raise to assess neural tension [42], knee flexion, while prone to assess rectus femoris length [46], or hip lateral rotation range of motion to predict whether a patient might benefit from manipulation [45]. The findings of the current study suggest that assessment of passive movements may be important for other reasons as well. An indirect assessment of relative stiffness demonstrated during *passive* HLR may be an important component of a clinical examination for someone with LBP. An individual who demonstrates movement of elements within the lumbopelvic region soon after the start of *passive* HLR also may do so during *active* HLR. This early movement of elements within the lumbopelvic region may occur regardless of whether the patients received instructions to eliminate motion in the lumbopelvic region during HLR. Because eliminating or delaying rotation of elements within the lumbopelvic region during HLR is associated with a decrease in LBP symptoms [14, 15], examining relative stiffness during *passive* HLR may provide useful information for the development of the most appropriate plan of care and prognosis for a low back pain problem. Determining who demonstrates rotation of elements within the lumbopelvic region soon after the start of *passive* HLR may help clinicians determine who will have a harder time modifying the movement pattern with simple instructions and tactile cues. These individuals may need a different type of intervention from that applied in the current study. They also may have a poorer prognosis than individuals who demonstrate later onset of motion in the lumbopelvic region during *passive* HLR and who may correct more easily with simple instructions and tactile cues. 

Furthermore, examination of motion during *passive *HLR appears to be more valuable than examination of motion during *active *HLR as a predictor of performance during the *active-instructed* condition. Although the RMI during both *passive *HLR and *active* HLR was significantly correlated with the RMI during the *active-instructed *HLR, the RMI during the *passive* condition (1) had a stronger relationship with the RMI during the *active-instructed *condition and (2) explained 76% of the variance in the RMI during the *active-instructed *condition without any further variance statistically explained by adding additional variables to the regression equation. The RMI during the *active* condition had a weaker relationship with the RMI during the *active-instructed *condition and statistically explained no additional variance beyond that already explained by the RMI during the *passive* condition. A clinician who only examines the RMI during the *active *condition may not receive the same information they would have received had they examined the RMI during the *passive* condition.

Relative stiffness that is demonstrated during *passive* HLR might be particularly important in people with LBP. In the current study, people with and people without LBP both demonstrated rotation of elements within the lumbopelvic region soon after the start of *passive *HLR (small RMI value); however, only people with LBP demonstrated a relationship between the RMI during the *passive* condition and the RMI during the *active-instructed* condition. It is unknown why people with LBP demonstrate similar movements during the *passive *and the *active-instructed *conditions and people without LBP do not. There are, however, a number of potential contributing factors. Prior studies have demonstrated potential differences between people with and people without LBP in trunk muscle strength [47–50] and elements of motor control, including muscle recruitment [51–53] and proprioception [54, 55]. Pain also may affect the ability for an individual with LBP to recruit trunk musculature [52, 56]. Because all of these factors may affect movement, all of these factors have the potential to affect an individual's ability to overcome passive relative stiffness during *active* HLR. For example, in the current study, a person who demonstrates a small RMI value during *passive* HLR is thought to be relatively stiffer passively in the hip region than in the lumbopelvic region. To overcome this stiffness and eliminate rotation of elements within the lumbopelvic region during active HLR, this person might need to activate appropriate trunk muscles to increase the active stiffness of the lumbopelvic region during the *active* HLR motion. Individuals without LBP may have the muscle strength and motor control necessary to activate the trunk musculature sufficiently to overcome the stiffness of the hip region. Alternatively, if an individual with LBP (1) has weak musculature, (2) has proprioceptive deficits, or (3) is unable to recruit musculature secondary to pain; the individual may not be able to recruit trunk musculature enough or at an appropriate time to overcome the stiffness of the hip region during the *active* HLR motion. Further study is necessary to determine which of these, or other factors, contribute to the relationship between the RMI during the *passive *condition and the RMI during the *active-instructed *condition in people with LBP. 

People without LBP also demonstrated a negative relationship between shank length and the RMI during the *active-instructed *condition, but people with LBP did not. Compared to a shorter shank, a longer shank may be heavier with a center of mass that is further from the axis of rotation. A longer shank, therefore, could contribute to greater torque at the hip joint, requiring greater trunk muscle recruitment to avoid rotation in the lumbopelvic region as the hip rotates. Thus, it is not surprising that there would be a relationship between shank length and the RMI during the *active-instructed* condition. What is surprising is that the relationship is only in people without LBP and not people with LBP. Although it is logical that shank length would be an important factor to consider, perhaps in people with LBP who may have decreased muscle strength [47–50] and differences in motor control [51–55] compared to people without LBP, other factors such as passive relative stiffness become more predictive of performance during the *active-instructed* condition. Further study is necessary to explore why the relationships between the RMI during the *active-instructed *condition and other factors are different between people with and people without LBP.

It is also interesting in the current study that the data from 4 people with LBP, but none without LBP, were removed from analyses because of their inability to relax the monitored trunk and limb muscles during the *passive* trials. We have not explored why these 4 individuals were unable to relax and can only speculate as to the significance of this information in the current study. However, we think the inability to relax is important in some people with LBP and warrants further study in its relationship to movement patterns and the ability to alter movement patterns following instructions. 

There are a few limitations of the current study. We did not directly measure passive or active stiffness of elements within the lumbopelvic region or hip region. Additionally, the instrumented methods used in the current study are not routinely available to clinicians. Despite these limitations, we think the findings of the current study are clinically applicable. Stiffness cannot be measured directly in the clinic. Thus, examining relative stiffness through indirect methods is more clinically applicable. Similarly, the instrumented methods employed in the current study may not be available in all clinics, but relative stiffness can be assessed visually by clinicians. Clinically, visual assessment of relative stiffness is defined as greater than 1/2 inch of movement of the pelvis during the first 50% of the HLR movement [57, 58]. Based on these criteria, relative stiffness is determined to be present or absent but is not quantified. The instrumented methods used in the current study allow us to quantify what is visually apparent, but not quantifiable by clinicians. Quantifying the motion allows us to study the variability in movement patterns as well as the change in those movement patterns following instruction. 

We also realize that generalizability of the results of the current study is limited. The current study examined only 1 movement task, HLR, in a small sample of young, relatively healthy individuals with a BMI < 30 and no serious comorbidities. These individuals reported chronic or recurrent low back pain and minimal levels of pain and disability. It is unknown whether similar results would be found in a larger sample of people that were older and more acutely involved, reported more comorbidities, or reported a higher level of pain or disability. It is also unknown if similar results would be found with other limb movement tests. Despite the limitations in generalizability of the current study, we believe the results of this study are important. To our knowledge, this is the first study examining the relationship between passive relative stiffness and how well people modify performance of *active* HLR following standardized instructions. We believe this study supports the need for further examination of whether the findings of this study can be replicated with other movement tasks and in a variety of different people with LBP.

## 5. Conclusion

Examination of the movement pattern demonstrated during *passive* HLR may be important to consider when instructing someone to modify motion during *active* HLR. In the current study, people with LBP who demonstrated rotation of elements within the lumbopelvic region shortly after the start of *passive* HLR were most likely to do so during *active-instructed* HLR, despite receiving instructions on how to eliminate motion in the lumbopelvic region during the active HLR motion. These individuals may have more difficulty correcting active HLR than people with LBP who demonstrate rotation of elements within the lumbopelvic region later during *passive* HLR and thus may have a poorer prognosis. Further study is necessary to identify whether the relationship between *passive* and *active*-*instructed* conditions exists with other movement tasks and with a variety of different people with LBP.

## Figures and Tables

**Table 1:** Subject characteristics.

	People with LBP (*n* = 15)	People without LBP (*n* = 20)
Gender	M = 7, F = 8	M = 10, F = 10
Age (years)	28.1 (7.2)	26.5 (5.9)
Weight (kg)	74.1 (10.1)	72.6 (7.6)
Height (cm)	173.5 (10.9)	171.4 (9.4)
Body mass index (kg/m^2^)	24.8 (3.0)	24.9 (2.8)
Spine length (cm)	48.1 (3.6)	46.8 (2.8)
Shank length (cm)	38.7 (3.1)	38.1 (3.1)
Passive hip lateral rotation* (degrees)	45.4 (10.0)	49.1 (7.2)
Femoral anteversion^†^ (degrees)	11.3 (5.5)	11.4 (5.5)
Generalized joint hypermobility^‡^ (0–9)	2.2 (3.2)	2.1 (2.5)
Relative motion index^§^, *active *condition	4.7 (3.6)	6.7 (4.0)
Relative motion index, *active-instructed *condition	11.4 (8.5)	18.2 (12.3)
Relative motion index, *passive *condition	3.7 (7.1)	4.8 (4.6)
Current pain score^*||*^ (0–10)	2.0 (1.2)	NA
Duration of LBP (years)	6.8 (3.3)	NA
Modified Oswestry Disability Index^¶^ (0–100%)	13.5 (9.5)	NA
Number of acute flare-ups in previous 12 months^#^	5.8 (4.3)	NA

Values expressed as mean (standard deviation).

*P* > 0.5 for all comparisons.

*Passive hip lateral rotation with pelvis stabilized, measured in prone with an inclinometer.

^†^Femoral anteversion measured with a goniometer using methods described by Ruwe et al. [37].

^‡^Generalized joint hypermobility measured with Beighton Hypermobility Scale [38].

^§^Relative motion index calculated as the amount of hip lateral rotation completed prior to the start of lumbopelvic rotation.

^||^Pain measured using a verbal numeric pain rating scale [32].

^¶^Disability measured using modified Oswestry Disability Index [31].

^#^A flare-up is defined as a period (usually a week or less) when back pain is markedly more severe than usual [30].

**Table 2:** Pearson product-moment correlations between the relative motion index (RMI) during the *active-instructed *condition and subject characteristics, clinical findings, and laboratory findings.

	RMI, *active-instructed* condition			
	People with LBP		People without LBP	
	Correlations	*P* value	Correlations	*P* value
Gender	**0.570**	**0.027**	0.273	0.244
Age	−0.115	0.682	−0.120	0.613
Weight	−0.164	0.560	−0.318	0.171
Height	−0.393	0.149	−0.307	0.188
Spine length	−0.457	0.087	−0.295	0.206
Shank length	−0.251	0.368	−0.445	**0.049**
Passive hip lateral rotation*	0.028	0.922	0.030	0.900
Femoral anteversion^†^	0.022	0.943	0.191	0.420
Generalized joint hypermobility^‡^	0.242	0.384	0.087	0.715
RMI^§^, *passive* condition	**0.873**	**<0.001**	0.144	0.544
RMI, *active* condition	**0.654**	**0.008**	0.229	0.331

Abbreviations: HLR, hip lateral rotation; RMI, relative motion index.

Significant correlations indicated in bold-face type.

*Passive hip lateral rotation with pelvis stabilized, measured in prone with an inclinometer.

^†^Femoral anteversion measured with a goniometer using methods described by Ruwe et al. [37].

^‡^Generalized joint hypermobility measured with Beighton Hypermobility Scale [38].

^§^Relative motion index calculated as the amount of HLR angular motion completed prior to the start of lumbopelvic motion.

**Table 3:** Pearson product-moment correlations between (1) the relative motion index (RMI) during the *active* condition, (2) the RMI during the *passive* condition, and (3) gender in people with LBP.

	RMI, *active condition *	RMI, *passive condition *	Gender
RMI,* active* condition	1.00		
RMI,* passive* condition	0.834*	1.00	
Gender	0.786^†^	0.663^†^	1.00

*P < 0.001; ^†^ *P* < 0.01.

Abbreviation: RMI: relative motion index calculated as the amount of HLR angular motion completed prior to the start of lumbopelvic motion.

**Table 4:** Results of hierarchical multiple regression analyses for people with LBP.

Predictor variables	Criterion variable RMI, *active-instructed* condition	
	*R* ^2^ change	*P*-value
RMI, *passive *condition	0.762	**<0.001**
RMI, *active *condition	0.018	0.344
Gender	0.006	0.595
Total *R* ^2^	0.786	**0.001**

Significant *R* ^2^ change indicated in bold-face type.

Abbreviations: RMI: relative motion index calculated as the amount of hip lateral rotation angular motion completed prior to the start of lumbopelvic motion.
